# Local Label Point Correction for Edge Detection of Overlapping Cervical Cells

**DOI:** 10.3389/fninf.2022.895290

**Published:** 2022-05-12

**Authors:** Jiawei Liu, Huijie Fan, Qiang Wang, Wentao Li, Yandong Tang, Danbo Wang, Mingyi Zhou, Li Chen

**Affiliations:** ^1^State Key Laboratory of Robotics, Shenyang Institute of Automation, Chinese Academy of Sciences, Shenyang, China; ^2^Institutes for Robotics and Intelligent Manufacturing, Chinese Academy of Sciences, Shenyang, China; ^3^University of Chinese Academy of Sciences, Beijing, China; ^4^Key Laboratory of Manufacturing Industrial Integrated, Shenyang University, Shenyang, China; ^5^Department of Gynecology, Cancer Hospital of China Medical University, Liaoning Cancer Hospital & Institute, Shenyang, China; ^6^Department of Pathology, Cancer Hospital of China Medical University, Liaoning Cancer Hospital & Institute, Shenyang, China

**Keywords:** label correction, point correction, edge detection, segmentation, local point smoothing, cervical cell dataset

## Abstract

Accurate labeling is essential for supervised deep learning methods. However, it is almost impossible to accurately and manually annotate thousands of images, which results in many labeling errors for most datasets. We proposes a local label point correction (LLPC) method to improve annotation quality for edge detection and image segmentation tasks. Our algorithm contains three steps: gradient-guided point correction, point interpolation, and local point smoothing. We correct the labels of object contours by moving the annotated points to the pixel gradient peaks. This can improve the edge localization accuracy, but it also causes unsmooth contours due to the interference of image noise. Therefore, we design a point smoothing method based on local linear fitting to smooth the corrected edge. To verify the effectiveness of our LLPC, we construct a largest overlapping cervical cell edge detection dataset (CCEDD) with higher precision label corrected by our label correction method. Our LLPC only needs to set three parameters, but yields 30–40% average precision improvement on multiple networks. The qualitative and quantitative experimental results show that our LLPC can improve the quality of manual labels and the accuracy of overlapping cell edge detection. We hope that our study will give a strong boost to the development of the label correction for edge detection and image segmentation. We will release the dataset and code at: https://github.com/nachifur/LLPC.

## 1. Introduction

Medical image datasets are generally annotated by professional physicians (Demner-Fushman et al., 2016; Almazroa et al., 2017; Johnson et al., 2019; Zhang et al., 2019; Lin et al., 2021; Ma et al., 2021; Wei et al., 2021). To construct an annotated dataset for edge detection or image segmentation tasks, annotators often need to annotate points and connect them into an object outline. In the manual labeling process, it is difficult to control label accuracy due to human error. Northcutt et al. (2021) found that label errors are numerous and universal: the average error rate in 10 datasets is 3.4%. These wrong labels seriously affect the accuracy of model evaluation and destabilize benchmarks, which will ultimately spill over model selection and deployment. For example, the deployed model in learning-based computer-aided diagnosis (Saha et al., 2019; Song et al., 2019, 2020; Wan et al., 2019; Zhang et al., 2020) is selected from many candidate models based on evaluation accuracy, which means that inaccurate annotations may ultimately affect accurate diagnosis. To mitigate labeling errors, an image is often annotated by multiple annotators (Arbelaez et al., 2010; Almazroa et al., 2017; Zhang et al., 2019), which generates multiple labels for one image. However, even if the annotation standard is unified, differences between different annotators are inevitable. Another way is to correct the labels manually (Ma et al., 2021). In fact, multi-person annotation and manual label correction are time-consuming and labor-intensive. Therefore, it is of great value to develop label correction methods based on manual annotation for supervised deep learning methods.

**Figure 1:**
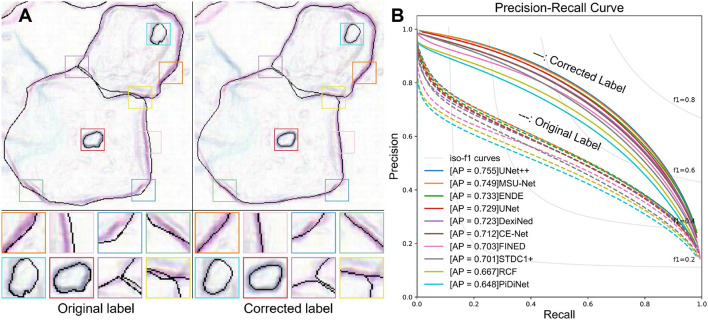
**(A)** Visual comparison of the original label and our corrected label. Our LLPC can improve the edge positioning accuracy and generate more accurate edge labels. **(B)** Precision-Recall curves of edge detection methods on our CCEDD dataset. The average precision (AP) is significantly improved over multiple baseline models by using our corrected labels.

Most label correction works are focused on weak supervision (Zheng et al., 2021), semi-supervision (Li et al., 2020), crowdsourced labeling (Bhadra and Hein, 2015; Nicholson et al., 2016), classification (Nicholson et al., 2015; Kremer et al., 2018; Guo et al., 2019; Liu et al., 2020; Wang et al., 2021; Li et al., 2022), and natural language processing (Zhu et al., 2019). However, label correction in these tasks is completely different from correcting object contours. To automatically correct edge labels, we propose a local label point correction method for edge detection and image segmentation. Our method contains three steps: gradient-guided point correction, point interpolation, and local point smoothing. We correct the annotation of the object contours by moving label points to the pixel gradient peaks and smoothing the edges formed by these points. To verify the effectiveness of our label correction method, we construct a cervical cell edge detection dataset. Experiments with multiple state-of-the-art deep learning models on the CCEDD show that our LLPC can greatly improve the quality of manual annotation and the accuracy of overlapping cell edge detection, as shown in Figure 1. Our unique contributions are summarized as follows:

- We are the first to propose a label correction method based on annotation points for edge detection and image segmentation. By correcting the position of these label points, our label correction method can generate higher-quality label, which contributes 30–40% AP improvement on multiple baseline models.
- We construct a largest publicly cervical cell edge detection dataset based on our LLPC. Our dataset is ten times larger than the previous datasets, which greatly facilitates the development of overlapping cell edge detection.
- We present the first publicly available label correction benchmark for improving contour annotation. Our study serves as a potential catalyst to promote label correction research and further paves the way to construct accurately annotated datasets for edge detection and image segmentation.

## 2. Related Work

### 2.1. Label Correction

Deep learning is developing rapidly with the help of big computing (Jouppi et al., 2017) and big data (Deng et al., 2009; Sun et al., 2017; Zhou et al., 2017). Some works (Radford et al., 2019; Brown et al., 2020; Raffel et al., 2020) focus on feeding larger models with more data for better performance and generalization, while others design task-specific model structures and loss functions (Hu et al., 2019; Huang et al., 2021; Zhao et al., 2022) to improve performance on a fixed dataset. Recently, data itself has received a lot of attention. Ng et al. (2021) led the data revolution of deep learning and successfully organized the first “Data-Centric AI” competition. The competition aims to improve data quality and develop data optimization pipelines, such as label correction, data synthesis, and data augmentation (Motamedi et al., 2021). Competitors mine data potential instead of optimizing model structure to improve performance. Northcutt et al. (2021) found that if the error rate of test labels only increases by 6%, ResNet18 outperforms ResNet-50 on ImageNet (Deng et al., 2009). To improve data quality and accurately evaluate models, there is an urgent need to develop label correction algorithms. In weak supervision and semi-supervision (Li et al., 2020; Zheng et al., 2021), pseudo label correction is usually implemented due to the lack of supervision from real labels. Zheng et al. (2021) correct the noisy labels by using a meta network for image recognition and text classification. For supervised learning, bad data can be discarded by data preprocessing, but bad labels seem inevitable in large-scale datasets. In crowdsourcing (Bhadra and Hein, 2015; Nicholson et al., 2016), an image is annotated by multiple people to improve the accuracy of classification task (Nicholson et al., 2015; Kremer et al., 2018; Guo et al., 2019). Guo et al. (2019) trained a model by using a small amount of data and design a label completion method to generate labels (negative or positive) for the mostly unlabeled data. However, label correction in these tasks is significantly different from correcting object contours. In this paper, to eliminate edge location errors and inter-annotator differences in manual annotation, we propose an label correction method based on annotation points for edge detection and image segmentation. Besides, we compare our LLPC with conditional random fields (CRF) (Sutton et al., 2012), which is popular as post-processing for other segmentation methods (Chen et al., 2017; Sun et al., 2020; Fan et al., 2021a; Lu et al., 2021; Ma et al., 2022; Zhang et al., 2022). Dense CRF (Krähenbühl and Koltun, 2011) improves the labeling accuracy by optimizing energy function based on coarse segmentation images, while our LLPC is a label correction method based on annotation points, which are two different technical routes of label correction for image segmentation. More discussion in Section 5.3.

### 2.2. Cervical Cell Dataset

Currently, cervical cell datasets include ISBI 2015 challenge dataset (Lu et al., 2015), Shenzhen University dataset (Song et al., 2016), and Beihang University dataset (Wan et al., 2019). Supervised deep learning based methods require large amounts of data with accurate annotations. However, the only public ISBI dataset (Lu et al., 2015) has a small amount of data and simple image types, which are difficult to train deep neural networks. In this paper, we construct a largest high-accuracy cervical cell edge detection dataset based on our label correction method. Our CCEDD contains overlapping cervical cell masses in a variety of complex backgrounds and high-precision corrected labels, which are sufficient in quantity and richness to train various deep learning models.

## 3. Label Correction

**Figure 2:**
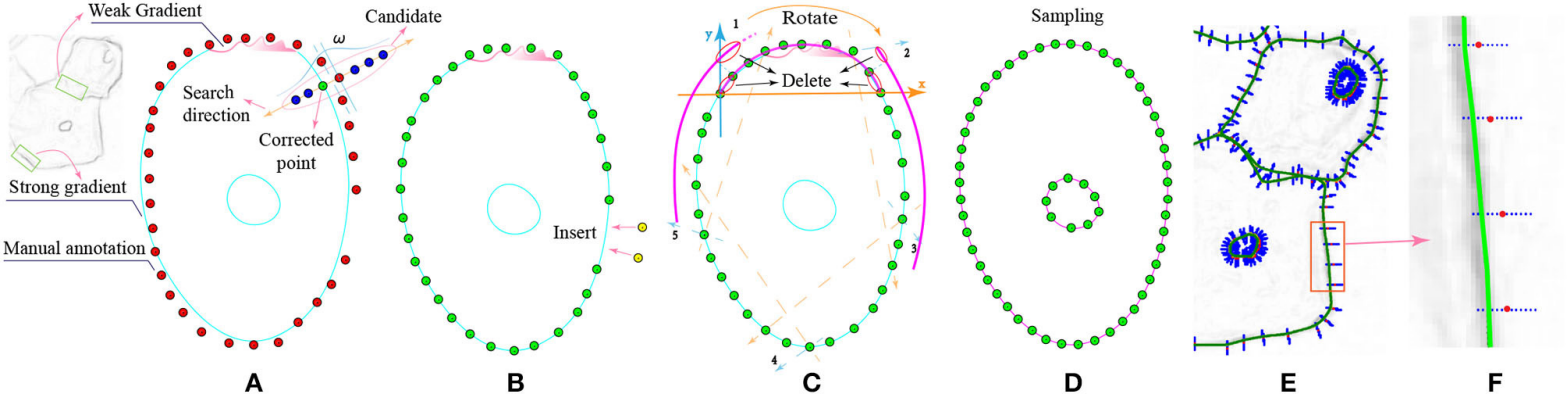
The workflow of our LLPC algorithm. **(A)** Gradient-guided point correction (the red points → the green points); **(B)** Insert points at large intervals; **(C)** Piecewise curve fitting (the purple curve); **(D)** Curve sampling; **(E)** The gradient image with the corrected edge label (the green edges); **(F)** Magnification of the gradient image. The whole label correction process is to generate the corrected edge (green edges) from original label points (red points) in **(F)**.

Our LLPC contains three steps: gradient-guided point correction (GPC), point interpolation (PI) and local point smoothing (LPS). *I*(*x, y*) is a cervical cell image and *g*(*x, y*) is the gradient image of *I*(*x, y*) after Gaussian smoothing. $x_{s}^{i}$ is an original label point of *I*(*x, y*). First, we correct the points $x_{s}^{i}$ to the nearest gradient peak on *g*(*x, y*), as shown in Figure 2A, i.e., $\left\{x_{s}^{i}\right\}\to \left\{x_{c}^{i}\right\}$. *i* ∈ {1, 2, …, *n*_*s*_}. Second, we insert more points in large gaps, as shown in Figure 2B, i.e., $\left\{x_{c}^{i}\right\}\;\to \left\{x_{I}^{j}\right\}$. *j* ∈ {1, 2, …, *n*_*I*_}. *n*_*s*_ and *n*_*I*_ are the number of points before and after interpolation, respectively. Third, we divide the point set $\left\{x_{I}^{j}\right\}$ into *n*_*c*_ groups. Each group of points is expressed as Φ_*k*_. We fit a curve *C*_*k*_ on Φ_*k*_. *k* ∈ {1, 2, …, *n*_*c*_}. All curves {*C*_*k*_} are merged into a closed curve *C*_*c*_, as shown in Figure 2C. Finally, we sample *C*_*c*_ to obtain discrete edges *C*_*d*_, as shown in Figure 2D. In fact, the closed discrete edges generated by multiple curves fusion are not smooth at the stitching nodes. Therefore, we propose a local point smoothing method without curves splicing and sampling in Section 3.3.

### 3.1. Gradient-Guided Point Correction

Although the annotations of cervical cell images are provided by professional cytologists, due to human error, the label points usually deviate from the pixel gradient peaks. To solve this problem, we design a gradient-guided point correction (GPC) method based on gradient guidance. We correct the label points only in the strong gradient region to eliminate human error, while preserving the original label points in the weak gradient region to retain the correct high-level semantics in human annotations. Our point correction consists of three steps as follows:

1. Determine whether the position of each label point is in strong gradient regions.
2. Select a set of candidate points for a label point.
3. Move the label point to the position of the point with the largest gradient value among these candidate points.

The processing object of our LLPC is a set of label points ($\left\{x_{s}^{i}\right\}$) corresponding to a closed contour. For an original label point $x_{s}^{i}$, we select candidate points along the normal direction of label edge, as shown in Figure 2A. These points constitute a candidate point set $\Omega_{x_{s}^{i}}$, and $x_{max}^{i}$ is the point with the largest gradient in $\Omega_{x_{s}^{i}}$. We move $x_{s}^{i}$ to the position of $x_{max}^{i}$ to obtain the corrected label point $x_{c}^{i}$.


(1) $$\begin{array}{l} x_{c}^{i}=\{\begin{array}{cc} x_{max}^{i} & if\;\Delta >0\\ x_{s}^{i} & otherwise \end{array} \end{array}$$

where


(2) $$\begin{array}{l} \Delta =\left|max\left(\omega_{j}\cdot g\left(x_{s_{j}}^{i}\right)\right)-min\left(\omega_{j}\cdot g\left(x_{s_{j}}^{i}\right)\right)\right|-\lambda_{t}\cdot max\left(\omega_{j}\right). \end{array}$$

$x_{s_{j}}^{i}$ is a candidate point in $\Omega_{x_{s}^{i}}$. We judge whether a point $x_{s}^{i}$ is in strong gradient regions through Δ. If Δ > 0, the point will be corrected; otherwise, it will not be moved. In this way, when the radius (*r*) of $\Omega_{x_{s}^{i}}$ is larger, our method can correct larger annotation errors. However, this will increase the correction error of label points due to image noise and interference from adjacent edges. To balance the contradiction, the gradient value of the candidate point $g\left(x_{s_{j}}^{i}\right)$ is weighted by ω_*j*_, which allows setting a larger radius to correct larger annotation errors. We compute the weight as


(3) $$\begin{array}{l} \omega_{j}=K\left({\left\|x_{s_{j}}^{i}-x_{s}^{i}\right\|}_{2},h_{1}\right), \end{array}$$

where


(4) $$\begin{array}{l} K\left(x,h\right)=\kappa \left(x/h\right)/h. \end{array}$$

*K*(*x, h*) is a weighted kernel function with bandwidth *h*. κ(*x*) is a Gaussian function with zero mean and one variance. After point correction, $\left\{x_{s}^{i}\right\}\to \left\{x_{c}^{i}\right\}$.

### 3.2. Piecewise Curve Fitting

The edge generated directly from the point set $\left\{x_{c}^{i}\right\}$ is not smooth due to the errors in point correction process (see Section 5.4). To eliminate the errors, we fit multiple curve segments and stitch them together. In the annotation process of manually drawing cell contours, the annotators perform dense point annotations near large curvatures, and sparse annotations near small curvatures to accurately and quickly outline cell contours. Since the existence of large intervals is not conducive to curve fitting, we perform linear point interpolation (PI) on these intervals before curve fitting.

#### 3.2.1. Point Interpolation

The sparse label point pairs can be represented as,


(5) $$\begin{array}{l} \left\{\left(x_{c}^{i},x_{c}^{i+1}\right)|{\left\|x_{c}^{i}-x_{c}^{i+1}\right\|}_{2}>2\cdot gap\right\}, \end{array}$$

where *i* = 0, 1…*n*_*s*_ − 1. Then, we insert points between the sparse points pairs to satisfy


(6) $$\begin{array}{l} {\left\|x_{I}^{j}-x_{I}^{j+1}\right\|}_{2}<gap, \end{array}$$

as shown in Figure 2B. *j* = 0, 1…*n*_*I*_ − 1. *n*_*s*_ and *n*_*I*_ are the number of points before and after interpolation, respectively. *gap* is the maximum interval between adjacent point pair. After interpolation, $\left\{x_{c}^{i}\right\}\;\to \left\{x_{I}^{j}\right\}$.

#### 3.2.2. Curve Fitting

**Figure 3:**
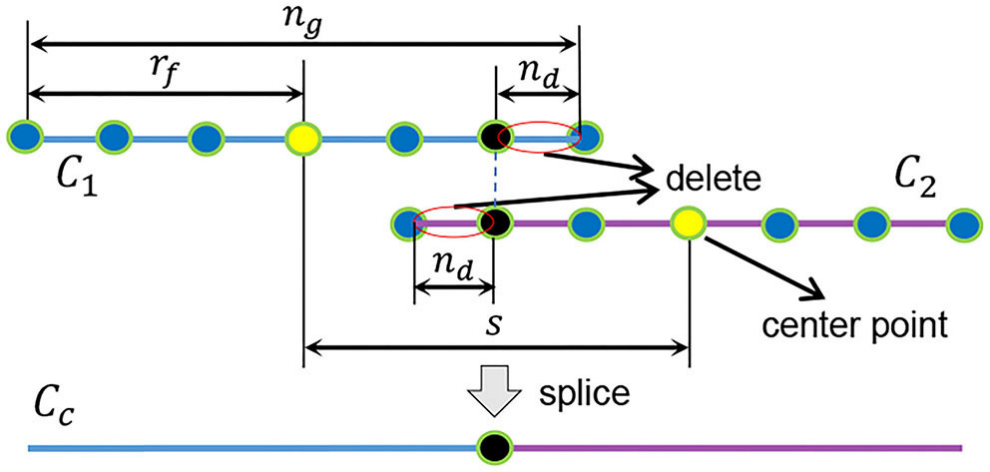
Merge multiple curves (*C*_1_ and *C*_2_) into one curve(*C*_*c*_).

We divide $\left\{x_{I}^{j}\right\}$ into *n*_*c*_ groups. Each group is expressed as $\Phi_{k}=\left\{x_{I}^{1+k\cdot s},x_{I}^{2+k\cdot s},\ldots ,x_{I}^{n_{g}+k\cdot s}\right\}$. *k* = 0, 1…*n*_*c*_ − 1. *n*_*c*_ = ⌈*n*_*I*_/*s*⌉. As shown in Figure 3, *s* = 2(*r*_*f*_ − *n*_*d*_) is the interval between the center points of each group; *r*_*f*_ = ⌊(*n*_*g*_ − 1)/2⌋ is the group radius; *n*_*g*_ is the number of points in the group. To reduce the fitting error at both ends of the curve, there is overlap between adjacent curves. The overlapping length is 2*n*_*d*_. To fit a curve on Φ_*k*_, we create a new coordinate system, as shown in Figure 2C. The x-axis passes through the $x_{I}^{1+k\cdot s}$ point and the $x_{I}^{n_{g}+k\cdot s}$ point. The point set in the new coordinate system is $\Phi_{k}^{r}$. We obtain a curve *C*_*k*_ by local linear fitting (McCrary, 2008) on $\Phi_{k}^{r}$. This is equivalent to solving the following problem at the target point *x*_*t*_ = (*x, y*) on the curve *C*_*k*_.


(7) $$\begin{array}{l} \underset{\beta_{0}\left(x\right),\beta_{1}\left(x\right)}{min}\overset{n_{g}+k\cdot s}{\underset{j=1+k\cdot s}{\sum }}\omega_{j}\left(x\right)\left(y_{j}-\beta_{0}\left(x\right)-\beta_{1}\left(x\right)\cdot x_{j}\right) \end{array}$$

β_0_(*x*) and β_1_(*x*) are the curve parameter at the point *x*_*t*_. (*x*_*j*_, *y*_*j*_) denotes the coordinates of point $x_{I}^{j}$ in $\Phi_{k}^{r}$. The weight function is


(8) $$\begin{array}{l} \omega_{j}\left(x\right)=K\left({\left\|x-x_{j}\right\|}_{2},h_{2}\right)/\overset{n_{g}+k\cdot s}{\underset{m=1+k\cdot s}{\sum }}K\left({\left\|x-x_{m}\right\|}_{2},h_{2}\right). \end{array}$$

If the distance between the point $x_{I}^{j}$ and the target point *x*_*t*_ is larger, the weight ω_*j*_(*x*) will be smaller. The matrix representation of the above parameter solution is


(9) $$\begin{array}{l} \beta ={\left(X^{T}\omega X\right)}^{-1}X^{T}\omega Y, \end{array}$$

where $X=\left[\begin{array}{cc} 1 & x_{1+k\cdot s}\\ 1 & x_{2+k\cdot s}\\ \vdots & \vdots \\ 1 & x_{n_{g}+k\cdot s} \end{array}\right]$, $Y=\left[\begin{array}{c} y_{1+k\cdot s}\\ y_{2+k\cdot s}\\ \vdots \\ y_{n_{g}+k\cdot s} \end{array}\right]$, $\beta =\left[\begin{array}{c} \beta_{0}\left(x\right)\\ \beta_{1}\left(x\right) \end{array}\right]$,


$$\omega =\left[\begin{array}{cccc} \omega_{1+k\cdot s}\left(x\right) & \; & \; & \;\\ \; & \omega_{2+k\cdot s}\left(x\right) & \; & \;\\ \; & \; & \ddots & \;\\ \; & \; & \; & \omega_{n_{g}+k\cdot s}\left(x\right) \end{array}\right].$$

The matrix ω is zero except for the diagonal. Each $\Phi_{k}^{r}$ corresponds to a curve *C*_*k*_. We stitch *n*_*c*_ curves into a closed curve *C*_*c*_, as shown in Figures 2C, 3. Then, we sample on the interval $\left[x_{I}^{1+k\cdot s+n_{d}},x_{I}^{n_{g}+k\cdot s-n_{d}}\right]$ as shown in Figure 2D. We convert the coordinates of these sampling points to the original image coordinate system. Finally, we can obtain a discrete edge *C*_*d*_, as shown in Figures 2E,F.

### 3.3. Local Point Smoothing

**Figure 4:**
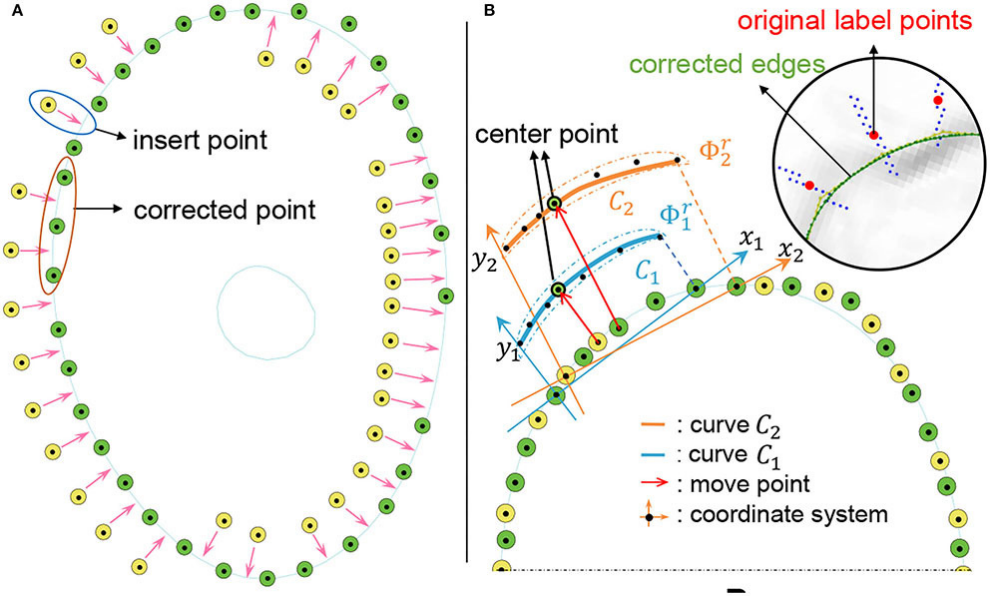
Local point smoothing to generate smooth closed discrete edges. **(A)** Insert points; **(B)** Move the coordinate system and correct each point by curve fitting. All corrected points constitute a discrete edge.

In Section 3.2, we stitch multi-segment curves to obtain a closed cell curve, and then sample the curve to generate a discrete edge. In fact, there is no smoothness at the splice nodes. To generate a smooth closed discrete edge, we design a local point smoothing (LPS) method without curves splicing and sampling. As shown in Figure 4A, we insert more points in large intervals (*gap* = 1). As shown in Figure 4B, we only correct the center point of $\Phi_{k}^{r}$ by fitting a curve (*C*_*k*_). By shifting the local coordinate system by one step (*s* = 1), each point in $\left\{x_{I}^{j}\right\}$ will be corrected by fitting a curve. These correction points constitute a discrete edge *C*_*d*_. Because no curves are spliced, the generated edge is smooth at each point. The pipeline of our LLPC is shown in Algorithm 1.

**Algorithm 1:**
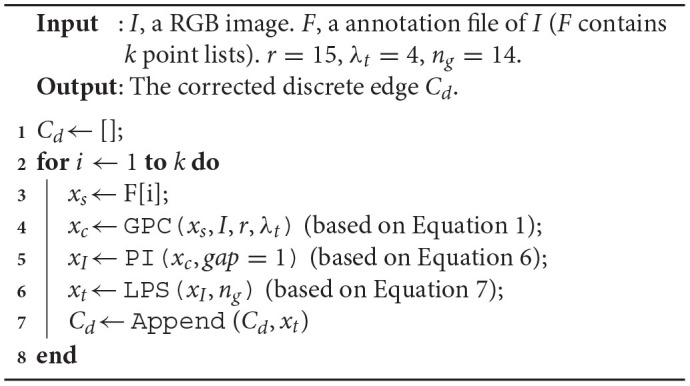
LLPC Label Correction Algorithm.

### 3.4. Parameter Setting

In Section 3.1, we set the parameters *r* = 15, λ_*t*_ = 4 and *h*_1_ = *r*/2. In Section 3.2, we set *n*_*g*_ = 14. *r*_*f*_ = ⌊(*n*_*g*_ − 1)/2⌋. *h*_2_ = *r*_*f*_/2. When *gap* = 1 and *s* = 1, **the Section** **3.3** **is a special case of the Section** **3.2**. See Section 5.4 for more discussion of parameter selection.

## 4. Experimental Design

### 4.1. Data Aquisition and Processing

We compare our CCEDD with other cervical cytology datasets in Table 1. Our dataset was collected from Liaoning Cancer Hospital & Institute between 2016 and 2017. We capture digital images with a Nikon ELIPSE Ci slide scanner, SmartV350D lens and a 3-megapixel digital camera. For patients with negative and positive cervical cancer, the optical magnification is 100× and 400×, respectively. All of the cases are anonymized. All processes of our research (image acquisition and processing, etc.) follow ethical principles. Our CCEDD dataset includes 686 cervical images with a size of 2,048×1,536 pixels (Table 2). Six expert cytologists outline the closed contours of the cytoplasm and nucleus in cervical cytological images by an annotation software (labelme; Wada, 2016).

**Table 1:** Comparison with other cervical cytology datasets.

**Dataset**	**Image size**	**Dataset size**	**Dataset size (512×512)**	**Open**
ISBI (Lu et al., 2015)	1,024×1,024	17	68	√
SZU Dataset (Song et al., 2016)	1,360×1,024	21	84	×
BHU Dataset (Wan et al., 2019)	512×512	580	580	×
CCEDD	**2,048×1,536**	**686**	**8,232**	**√**

*For a fair comparison of the sizes of different datasets, we crop the images to 512×512, and our CCEDD is about ten times larger than other datasets. Best results are highlighted*.

**Table 2:** The detailed description of CCEDD.

**Our CCEDD**	**Uncut CCEDD**	**Cut CCEDD**
Image size	2,048×1,536	512×384
Training set size	411	20,139
Validation set size	68	3,332
Test set size	207	10,143
Dataset size	686	33,614

**Figure 5:**
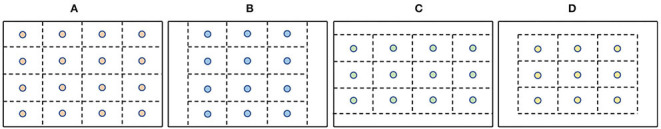
Image cutting method. **(A)** 4×4 cutting grid; **(B)** move the grid right; **(C)** move the grid down; **(D)** move the grid right and down.

We randomly shuffle our dataset and split it into training, validation and test sets. To ensure test reliability, we set this ratio to 6:1:3. To be able to train various complex neural networks on a GPU, we crop a large-size image into small-size images. If an image is cut as shown in Figure 5A, it will result in incomplete edge at the cut boundary. To maximize data utilization efficiency, we move the cutting grid, as shown in Figures 5B–D. After label correction, we cut an image with a size of 2,048×1,536 into 49 image patches with a size of 512×384 pixels.

### 4.2. Baseline Model and Evaluation Metrics

#### 4.2.1. Baseline Model

Our baseline detectors are 10 state-of-the-art models. We evaluate multiple edge detectors, such as RCF (Liu et al., 2019), ENDE (Nazeri et al., 2019), DexiNed (Poma et al., 2020), FINED (Wibisono and Hang, 2020), and PiDiNet (Su et al., 2021b). Furthermore, we explore more network structures for edge detection by introducing segmentation networks, which usually only requires simple modifications of the last layer of networks. These segmentation networks include STDC (Fan et al., 2021b), UNet (Ronneberger et al., 2015), UNet++ (Zhou et al., 2019), CENet (Gu et al., 2019), MSU-Net (Su et al., 2021a). To aggregate more shallow features for edge detection, we modify multiple layers of STDC, i.e., STDC+. More details of these network structure can be found in our code implementation.

#### 4.2.2. Evaluation Metrics

We quantitatively evaluate the edge detection accuracy by calculating three standard measures (ODS, OIS, and AP) (Arbelaez et al., 2010). The average precision (AP) is the area under the precision-recall curve (Figure 1B). F1-score$=\frac{2\cdot precision\cdot recall}{precision+recall}$ is the harmonic average of precision and recall. ODS is the best F1-score for a fixed scale, while OIS is the F1-score for the best scale in each image.

### 4.3. Experimental Setup

#### 4.3.1. Training Strategy

Data augmentation can improve model generalization and performance (Bloice et al., 2019). In training, we perform rotation and shearing operations, which require padding zero pixels around an image. In testing, there is no zero pixel padding. This lead to different distributions of training and testing sets and degrade the model performance. Therefore, we perform data augmentation in pre-training and no augmentation during fine-tuning.

Due to the different structures and parameters of baseline networks, a fixed number of training iterations may lead to overfitting or underfitting. For accurate evaluation, we adaptively adjust the iteration number by evaluating the average accuracy (AP) on the validation set. The period of model evaluation is set 1 epoch for pre-training and 0.1 epoch for fine-tuning. After the *i*-th model evaluation, we can obtain *Model*_*i*_ and *AP*_*i*_ (*i* = 1, 2, ⋯ , 50). If *AP*_*i*_ < *min*(*AP*_*i*−*j*_), the training ends and we obtain the optimal model *Model*_*j*|*max*(*AP*_*j*_)_. *j* = 1, 2, 3 in pre-training and *j* = 1, 2, ⋯ , 10 in fine-tuning. The maximum iteration number is 50 epochs for pre-training and fine-tuning. Besides, we also dynamically adjust the learning rate to improve performance. The learning rate *l* decays from 1^−4^ to 1^−5^. If *AP*_*i*_ < *AP*_*i*−1_, *l*_*i*_ = *l*_*i*−1_/2.

#### 4.3.2. Implementation Details

We use the Adam optimizer (Kingma and Ba, 2015) to optimize all baseline networks on PyTorch (β_1_ = 0, β_2_ = 0.9). We use random normal initialization to initialize these networks. To be able to train various complex neural networks on a GPU, we resize the image to 256×192. The batch size is set 4. We perform color adjustment, affine transformation and elastic deformation for data augmentation (Bloice et al., 2019). All experiments are implemented on a workstation equipped with a Intel Xeon Silver 4110 CPUs and a NVIDIA RTX 3090 GPU.

## 5. Experimental Results and Discussion

### 5.1. Edge Detection of Overlapping Cervical Cells

**Figure 6:**
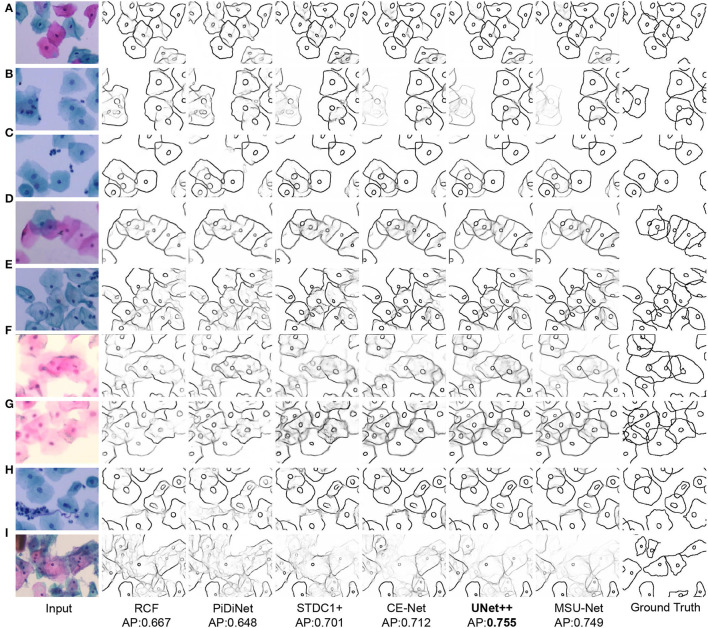
Visual comparison results on CCEDD dataset. **(A)** Slightly overlapping cells. **(B,C)** Highly overlapping cells. **(D,E)** Overlapping cell masses. **(F,G)** Blurred overlapping cells. **(H,I)** Overlapping cells in complex environments.

We show the visual comparison results on our CCEDD in Figure 6. The quantitative comparison is shown in Table 4 and Figure 1B. These results have important guiding implications for accurate edge detection of overlapping cervical cells. We analyze several factors affecting the performance of overlapping edge detection.

**Figure 7:**
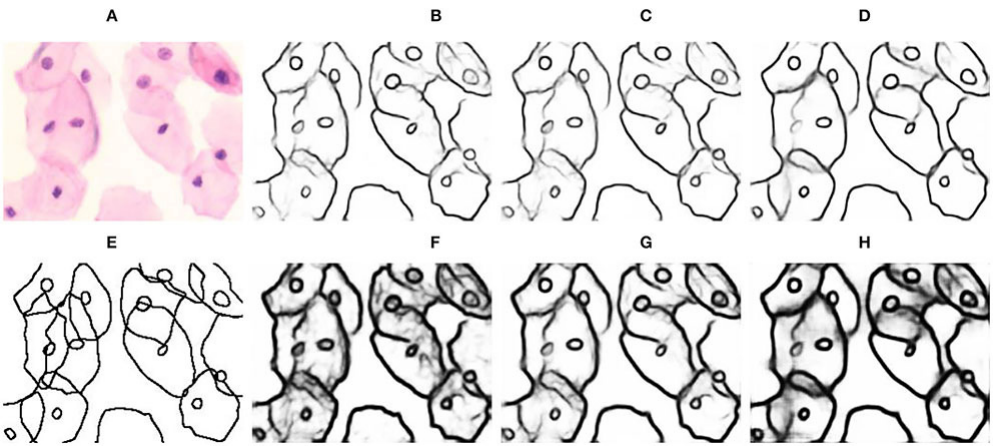
Visual comparison of different loss functions. **(A)** Input; **(E)** Ground truth; **(B,F)** PiDiNet (Su et al., 2021b); **(C,G)** RCF (Liu et al., 2019); **(D,H)** DexiNed (Poma et al., 2020); **(B–D)** BCELoss; **(F–H)** RCFLoss (Liu et al., 2019). “BCELoss” is binary cross entropy loss function. Compared with BCELoss, RCFLoss (Liu et al., 2019) can produce coarser edges.

- Loss function design. RCFLoss (Liu et al., 2019) produces coarser edges, as shown in Figure 7. This may be robust for natural images, but poor localization accuracy for accurate cervical cell edge detection.
- Network structure design. Long-distance skip connections can fuse shallow and deep features for constructing multi-scale features. Our experiments show that the U-shaped structure is effective for overlapping edge detection [e.g., UNet (Ronneberger et al., 2015), UNet++ (Zhou et al., 2019) and MSU-Net (Su et al., 2021a)].
- Pre-training. Due to the huge distribution difference between natural and medical images, pre-training may degrade performance (e.g., CE-Net; Gu et al., 2019) or have limited improvement (e.g., STDC; Fan et al., 2021b).

### 5.2. Effectiveness of Label Correction

**Figure 8:**
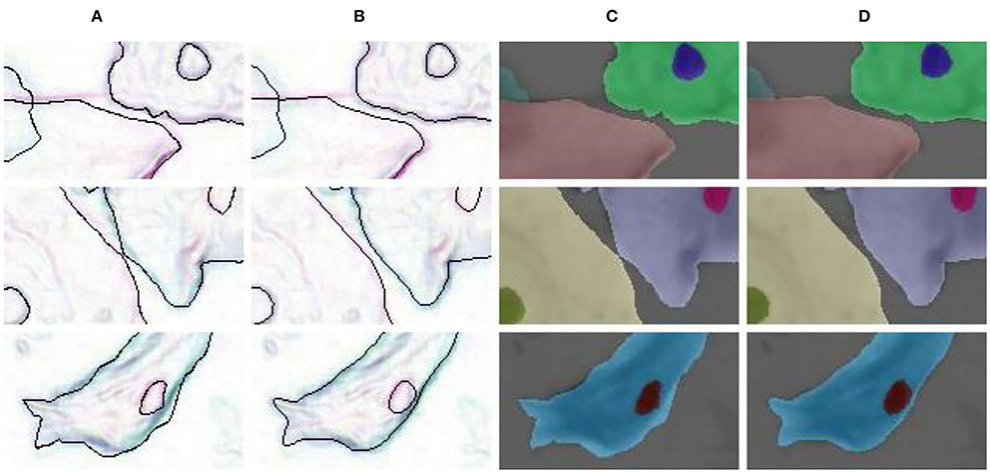
Label correction for edge detection and semantic segmentation. **(A)** Original edge; **(B)** Corrected edge; **(C)** Original mask; **(D)** Corrected mask.

In our LLPC, the position of label points is locally corrected to the pixel gradient peak. As shown in Figures 1A, 8B, Our LLPC can generate more accurate edge labels. Besides, we can easily generate corrected masks from corrected points in the labelme software (Wada, 2016). Compared with the original mask in Figure 8C, our corrected mask has higher edge localization accuracy and smoother edges, as shown in Figure 8D.

**Figure 9:**
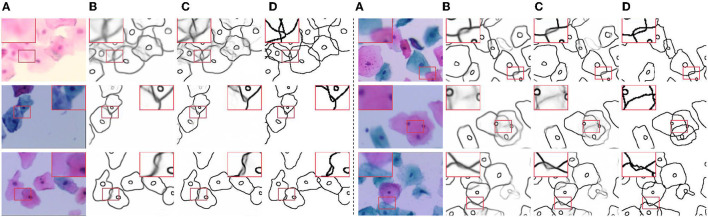
Visual comparison results of training with different labels. **(A)** Input image; **(B)** UNet++ (Zhou et al., 2019)/BCELoss + Original label; **(C)** UNet++ (Zhou et al., 2019)/BCELoss + Corrected label; **(D)** Corrected labels. Compared with the original label, the corrected label can improve the accuracy of overlapping edge detection.

We train multiple networks using original label and corrected label. The quantitative comparison results is shown in Table 3 and Figure 1B. Compared with the original label, using the corrected label to train multiple networks can significantly improve AP (**30–40**%), which verifies the effectiveness of our label correction method. Table 4 shows that the performance improvement comes from two aspects. First, our corrected label can improve the evaluation accuracy in testing (0.541 → 0.588). Second, using our corrected label to train network can improve the accuracy of overlapping edge detection in training (0.588 → 0.755), as shown in Figure 9.

**Table 3:** Edge detection results on our CCEDD dataset.

**Year/Model/Loss**	**ΔAP(%)**	**Label correction**			**No label correction**			**Params (M)**	**MACs(G)**
		**AP**	**ODS**	**OIS**	**AP**	**ODS**	**OIS**		
2019/RCF/RCFLoss	41.0	0.612	0.599	0.594	0.434	0.485	0.485	14.81	19.56
2019/RCF/BCELoss	41.9	0.667	0.638	0.645	0.470	0.507	0.512		
2019/ENDE/BCELoss	37.0	0.733	0.682	0.691	0.535	0.548	0.555	6.06	32.51
2020/DexiNed/RCFLoss	30.3	0.649	0.633	0.635	0.498	0.528	0.533	35.08	27.72
2020/DexiNed/BCELoss	38.5	0.723	0.671	0.680	0.522	0.541	0.549		
2020/FINED/RCFLoss	28.4	0.602	0.604	0.450	0.469	0.510	0.402	1.43	14.38
2020/FINED/BCELoss	41.4	0.703	0.660	0.621	0.497	0.528	0.530		
2021/PiDiNet/RCFLoss	37.2	0.590	0.581	0.574	0.430	0.481	0.479	**0.69**	**3.74**
2021/PiDiNet/BCELoss	**42.7**	0.648	0.624	0.628	0.454	0.496	0.501		
2021/STDC1/BCELoss	12.9	0.394	0.466	0.472	0.349	0.438	0.443	14.26	4.48
2021/STDC1(pretrain)/BCELoss	13.1	0.407	0.478	0.483	0.360	0.451	0.454		
2021/STDC2/BCELoss	16.1	0.403	0.473	0.478	0.347	0.435	0.442	22.30	7.01
2021/STDC2(pretrain)/BCELoss	15.0	0.413	0.484	0.488	0.359	0.449	0.454		
2021/STDC1+/BCELoss	41.3	0.701	0.652	0.659	0.496	0.518	0.524	13.76	39.28
2021/STDC2+/BCELoss	38.2	0.694	0.648	0.656	0.502	0.525	0.532	21.83	41.81
2015/UNet/BCELoss	38.9	0.729	0.679	0.689	0.525	0.539	0.546	31.03	41.96
2019/CE-Net(pretrain)/BCELoss	37.5	0.696	0.653	0.658	0.506	0.530	0.535	60.24	17.36
2019/CE-Net/BCELoss	36.4	0.712	0.668	0.675	0.522	0.540	0.547		
2019/UNet++(DS)/BCELoss	37.6	0.739	0.687	0.696	0.537	0.548	0.555	9.16	26.76
2019/UNet++/BCELoss	39.6	**0.755**	**0.691**	**0.701**	**0.541**	**0.550**	**0.557**		26.75
2021/MSU-Net/BCELoss	39.7	0.749	0.689	0.699	0.536	**0.550**	0.556	47.09	59.93

*Our baseline model contains RCF (Liu et al., 2019), ENDE (Nazeri et al., 2019), DexiNed (Poma et al., 2020), FINED (Wibisono and Hang, 2020), PiDiNet (Su et al., 2021b), STDC (Fan et al., 2021b), UNet (Ronneberger et al., 2015), CE-Net (Gu et al., 2019), UNet++ (Zhou et al., 2019), MSU-Net (Su et al., 2021a). “BCELoss” is binary cross entropy loss function. “RCFLoss” is an annotator-robust loss function for edge detection (Liu et al., 2019). STDC2 (Fan et al., 2021b) has more parameters than STDC1 (Fan et al., 2021b). “UNet++(DS)” is UNet++ (Zhou et al., 2019) with deep supervision. “MACs” is multiply-accumulate operation. “Params” and “MACs” are calculated by THOP^1^. Best and second best results are **highlighted** and underlined*.

**Table 4:** Performance improvement analysis of label correction.

**Training/Evaluation**	**AP**	**ODS**	**OIS**
Original label/Original label	0.541	0.550	0.557
Original label/Corrected label	0.588	0.592	0.598
Corrected label/Corrected label	**0.755**	**0.691**	**0.701**

*Use UNet++ (Zhou et al., 2019) for evaluation. Best results are highlighted*.

### 5.3. Comparison With Other Label Correction Methods

**Figure 10:**
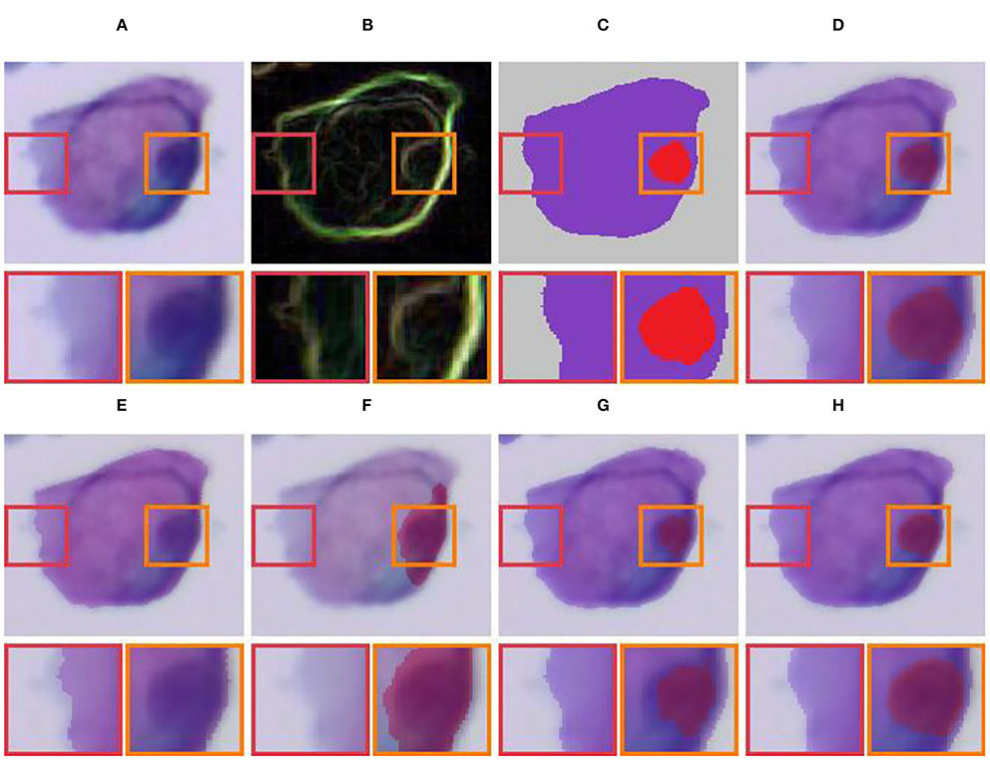
Qualitative comparison of single-cell label correction. **(A)** Input; **(B)** Gradient image; **(C)** Original mask; **(D)** Input + original mask; **(E)** Active contours (Chan and Vese, 2001) for cytoplasm; **(F)** Active contours (Chan and Vese, 2001) for nucleus; **(G)** Dense CRF (Krähenbühl and Koltun, 2011); **(H)** Our LLPC.

**Figure 11:**
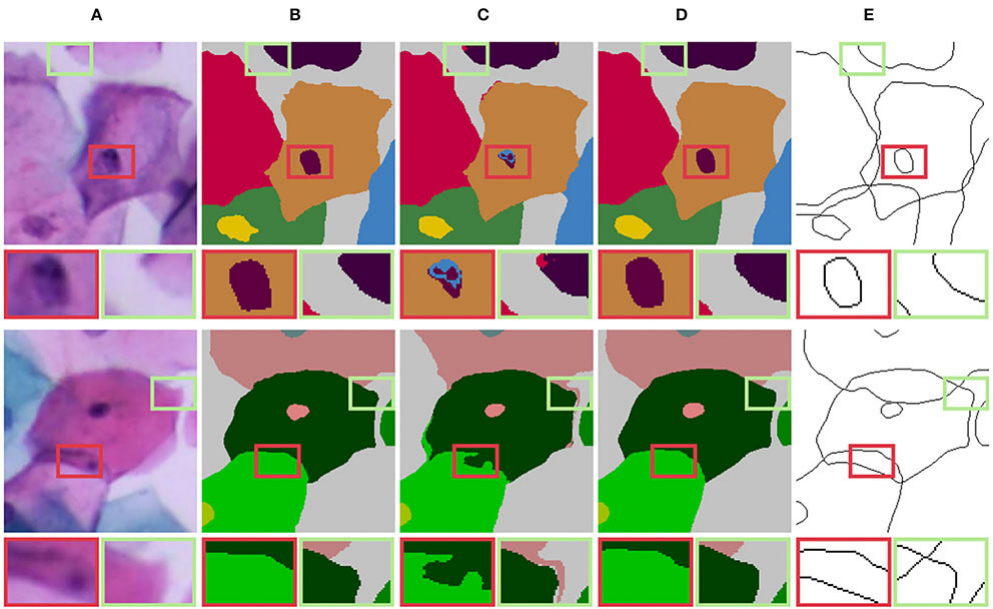
Qualitative comparison of label correction for overlapping cell masses. **(A)** Input; **(B)** Original mask; **(C)** Dense CRF (Krähenbühl and Koltun, 2011); **(D)** Our LLPC (mask); **(E)** Our LLPC (edge).

In Figures 10, 11, we compare our LLPC with active contours (Chan and Vese, 2001) and dense CRF (Krähenbühl and Koltun, 2011). We observed that active contours (Chan and Vese, 2001) is refinement failure of nucleus contours in Figure 10F, and dense CRF (Krähenbühl and Koltun, 2011) fails due to complex overlapping cell contours in Figure 11C. Since active contours (Chan and Vese, 2001) and dense CRF (Krähenbühl and Koltun, 2011) are global iterative optimization methods based on segmented images, which are uncontrollable for label correction of object contours and ultimately lead to these failed results. Our LLPC is the local label point correction without iterative optimization. Therefore, the correction error of our LLPC is controllable and the error in one place does not spread to other places, which is crucial for robust label correction. Besides, dense CRF (Krähenbühl and Koltun, 2011) is nonplussed over overlapping instance segmentation refinement, while our LLPC corrects label based on annotation point and can handle overlapping label correction, as shown in Figure 11E.

### 5.4. Ablation Experiment

#### 5.4.1. Ablation of Label Correction Method

**Figure 12:**
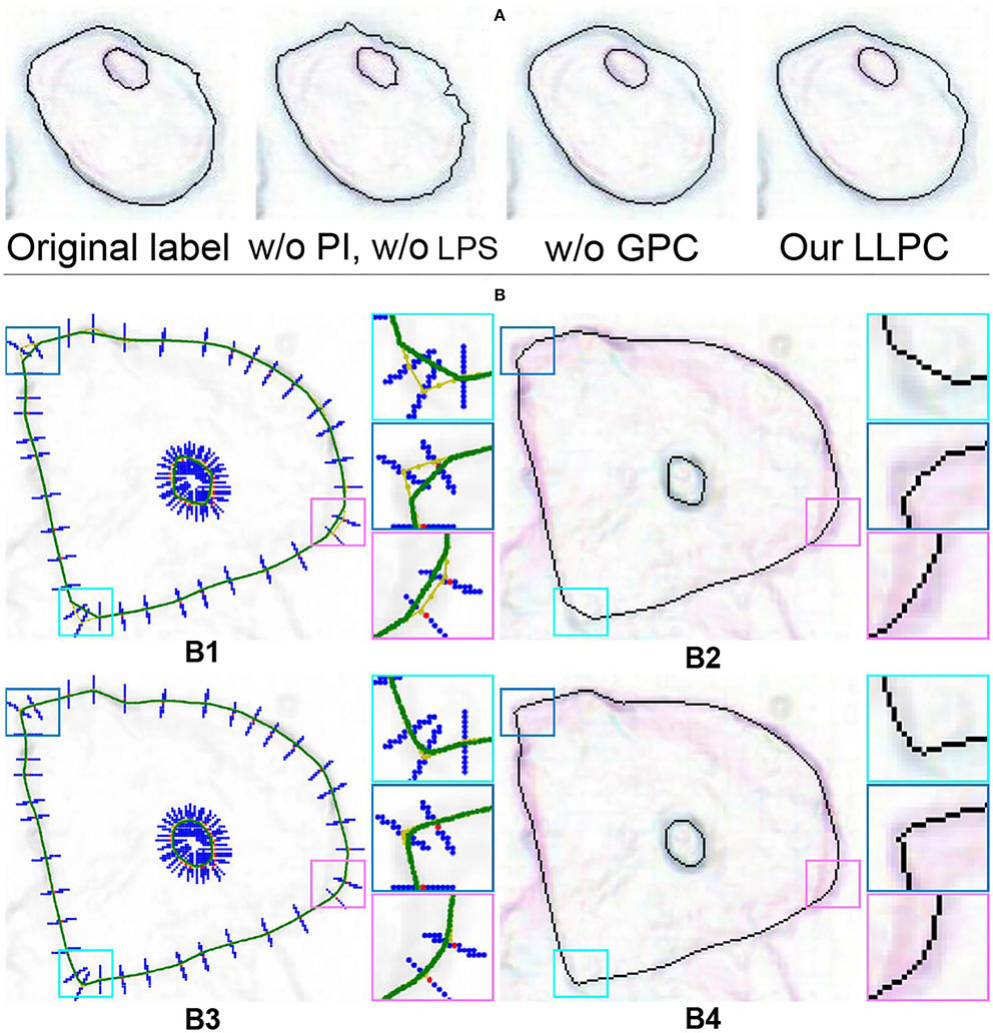
**(A)** Ablation of gradient-guided point correction. “GPC” is gradient-guided point correction. **(B)** Visual comparison of different label correction methods. (B1) The green curves generated by piecewise curve fitting (w/o LPS); (B2) The discrete edge sampled from the curves in (B1); (B3) The curves smoothed by LPS; (B4) The discrete edges without curve sampling.

Our LLPC contains three steps: gradient-guided point correction (GPC), point interpolation (PI), and local point smoothing (LPS). Although our GPC can correct label points to pixel gradient peaks, there is still some error in the correction process. LPS can smooth the edges corrected by GPC, as shown in Figure 12A. Table 5 shows that GPC is the most important part of our LLPC (0.541 → 0.731), while PI and LPS can further improve the annotation quality by smoothing edges (0.731 → 0.755). Only smoothing the original labels (“w/o GPC”) is ineffective (0.541 → 0.533). Because this may lead to larger annotation errors. Compared to piecewise curve fitting in Section 3.2, LPS can generate smoother edges, as shown in Figure 12B. These qualitative and quantitative results verify that the three components of our LLPC are essential.

**Table 5:** Ablation of our LLPC. “GPC” is gradient-guided point correction.

**Correction method**	**AP**	**ODS**	**OIS**
Original label	0.541	0.550	0.557
GPC (w/o PI, w/o LPS)	0.731	0.682	0.692
**Our LLPC**	**0.755**	**0.691**	**0.701**
w/o GPC	0.533	0.545	0.552
w/o PI	0.663	0.619	0.625
w/o LPS	0.742	0.689	0.699

“*w/o LPS” is using piecewise curve fitting instead of local point smoothing. Use UNet++ (Zhou et al., 2019) for evaluation. “PI” is point interpolation. Best results are highlighted*.

#### 5.4.2. Selection of Hyper-Parameters

**Figure 13:**
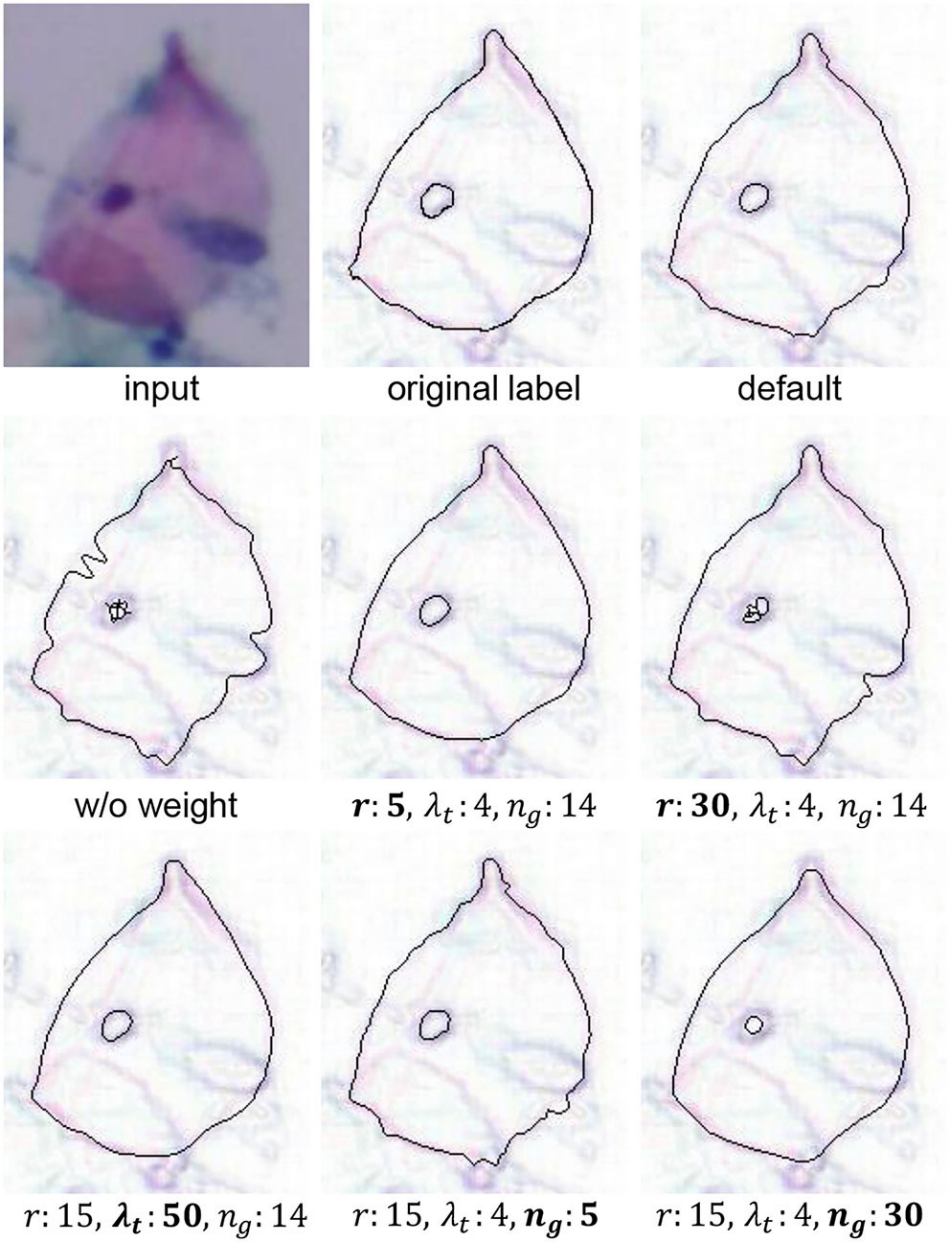
Visual comparison results for different parameters settings in our LLPC. “Default” is *r* = 15, λ_*t*_ = 4, and *n*_*g*_ = 14. “w/o weight” is ω_*j*_ = 1 in Equation (3).

To set the optimal parameters, we conduct parameters ablation experiments in Table 6. *gap* can control the point density in PI. For local curve fitting, *gap* = 1 is optimal. Therefore, for an unknown dataset, our LLPC only needs to set three parameters, i.e., *r*, λ_*t*_ and *n*_*g*_. A qualitative comparison of these parameters with different settings is shown in Figure 13. *r* controls the maximum error correction range in human annotations. If *r* is too small, large label errors cannot be corrected. If *r* is too large, the error of point correction is larger. *r* limits the correction range in space, while λ_*t*_ is the threshold for a limitation of gradient values variation during the correction process. If λ_*t*_ is large, label points are corrected only when the gradient value changes sharply in the search direction. *n*_*g*_ controls the scale of the local smoothing. For our CCEDD, *r* = 15, λ_*t*_ = 4, and *n*_*g*_ = 14.

**Table 6:** Parameters ablation of our label correction method.

**r**	**λ_*t*_**	**gap**	***n*_***g***_**	**AP**	**ODS**	**OIS**
7	4	1	14	0.691	0.645	0.653
11	4	1	14	0.732	0.681	0.691
19	4	1	14	0.746	0.691	0.701
23	4	1	14	0.734	0.683	0.692
15	1	1	14	0.750	0.689	0.700
15	2	1	14	0.751	0.690	0.700
15	3	1	14	0.745	0.691	0.700
15	5	1	14	0.750	0.689	0.699
15	10	1	14	0.729	0.679	0.688
15	15	1	14	0.708	0.658	0.664
15	4	1.5	14	0.749	0.688	0.698
15	4	2	14	0.742	0.689	0.699
15	4	1	10	0.750	0.689	0.699
15	4	1	12	0.729	0.687	0.697
15	4	1	16	0.752	0.690	0.700
15	4	1	18	0.750	0.687	0.698
15	4	1	14	**0.755**	**0.691**	**0.701**

*Use UNet++ (Zhou et al., 2019) for evaluation. For our CCEDD, we set r = 15, λ_t_ = 4, and n_g_ = 14. Best results are highlighted*.

#### 5.4.3. Ablation of Training Strategy

**Figure 14:**
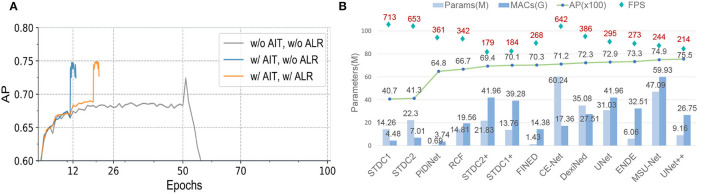
**(A)** Training schedules. “AIT” is adaptive iteration training. “ALR” is adaptive learning rate. We evaluate UNet++ (Zhou et al., 2019) on the validation set. **(B)** Comparison of network parameters, running efficiency and edge detection performance. “MACs” is multiply-accumulate operation. “FPS” is the average speed by evaluating 10,413 images with a resolution of 256×192.

Our training strategy can eliminate the influence of different distributions of the training and test sets due to data augmentation, and improve the AP by 3.6% in Table 7. To fairly evaluate multiple networks with different structures and parameters, we employ adaptive iteration and learning rate adjustment to avoid overfitting and underfitting. Table 8 and Figure 14A verify the effectiveness of our adaptive training strategy.

**Table 7:** Ablation of two-stage training strategy.

**Training methods**	**AP**	**ODS**	**OIS**
w/o augmentation, w/o fine-tuning	0.729	0.672	0.683
w/ augmentation, w/o fine-tuning	0.732	0.674	0.682
w/ augmentation, w/ fine-tuning	**0.755**	**0.691**	**0.701**

*We perform data augmentation in pre-training and no augmentation during fine-tuning. Use UNet++ (Zhou et al., 2019) for evaluation. Best results are highlighted*.

**Table 8:** Ablation of adaptive training strategy.

**Training methods**	**AP**	**ODS**	**OIS**	**epoch**
w/o AIT, w/o ALR	0.683	0.639	0.642	50
w/o AIT, w/o ALR	0.449	0.653	0.657	70
w/o AIT, w/o ALR	0.308	0.647	0.653	100
w/ AIT, w/o ALR	0.747	0.684	0.693	**13**
w/ AIT, w/ ALR	**0.750**	**0.693**	**0.700**	21

*We evaluate UNet++ (Zhou et al., 2019) on the validation set. “AIT” is adaptive iteration training. “ALR” is adaptive learning rate. Best results are highlighted*.

### 5.5. Computational Complexity

#### 5.5.1. Label Correction

Our LLPC takes 270 s to generate 100 corrected edge images with a size of 2,048×1,536 pixels on CPU. Because our label correction algorithm is offline and does not affect the inference time of a neural network, we have not further optimized it. If the algorithm runs on GPU, the speed can be further improved, which can save more time for label correction of large-scale datasets.

#### 5.5.2. Model Evaluation

We rewrite the evaluation code (Arbelaez et al., 2010) on GPU for fast evaluation. The average FPS using the UNet++ (Zhou et al., 2019) is 173 for 10,143 test images with a size of 256×192 pixels. In training, we need to calculate the AP of the validation set to adaptively control the learning rate and the number of iterations (see Section 4.3). Fast evaluation greatly accelerates our training process.

#### 5.5.3. Neural Network Inference

We test the inference speed of UNet++ (Zhou et al., 2019). For 207 images with a resolution of 1,024×768, the average FPS is 9. For 207 images with a resolution of 512×512, the average FPS is 26. For 10,413 images with a resolution of 256×192, the average FPS is 295. Figure 14B shows the running efficiency comparison of multiple benchmark models. According to the report of Wan et al. (2019), the methods of Wan et al. (2019), Lu et al. (2015), and Lu et al. (2016), took 17.67, 35.69m and 213.62 s for an image a resolution of 512×512, respectively. Compared with these method, the UNet++ (Zhou et al., 2019) is significantly faster. Many cervical cell segmentation approaches (Phoulady et al., 2017; Tareef et al., 2017, 2018; Wan et al., 2019; Zhang et al., 2020) consist of three stages, including nucleus candidate detection, cell localizations, and cytoplasm segmentation. Fast edge detection of overlapping cervical cell means that the detected edges can be used as a priori input of these segmentation networks to improve performance at a small cost.

## 6. Discussion

### 6.1. Label Correction for Natural Images

**Figure 15:**
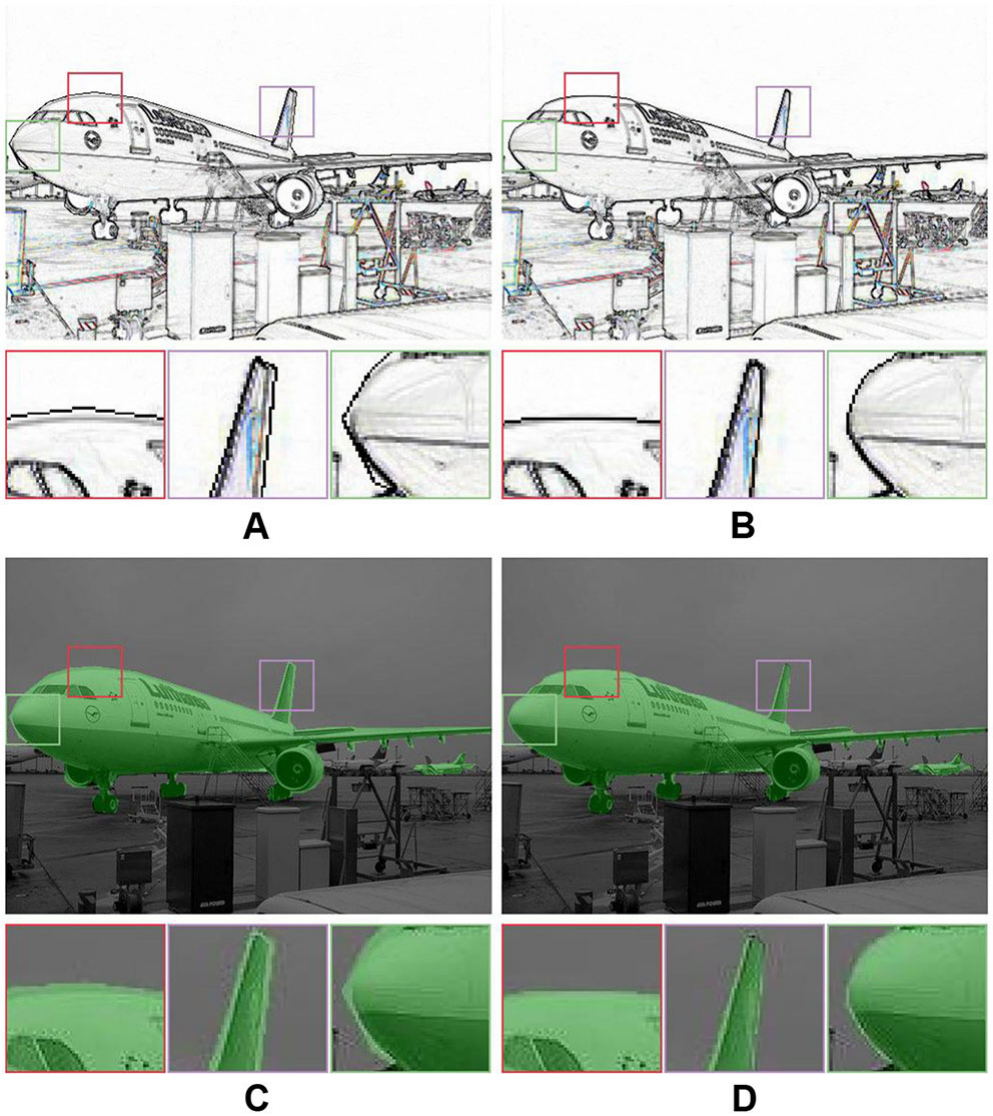
Label correction for natural images. **(A)** Original edge; **(B)** Corrected edge; **(C)** Original mask; **(D)** Corrected mask.

Our label correction method can correct a closed contour by correcting the position of label points, which does not require additional prior assumptions (e.g., contour shape, object size). We annotated several images in the PASCAL VOC dataset (Everingham et al., 2010) with labelme (Wada, 2016) and corrected the label (*r* = 7, λ_*t*_ = 4, and *n*_*g*_ = 9). As shown in Figure 15, our label correction method can generate more accurate object contours, which demonstrates the feasibility of our label correction method for natural images.

### 6.2. Overlapping Edge Detection

Overlapping edge detection of cervical cell is a challenging task due to the presence of strong and weak gradient edges. For edges with strong gradients, it only requires low-level detail features. For edges with weak gradients in overlapping region, it may require high-level semantics to reason contours and connect edges based on the context in strong gradient regions. While Unet++ (Zhou et al., 2019) achieves the best results on our CCEDD, there is no difference in the detection of these two different types of edges. Designing new network structures and loss functions for overlapping edge detection may be a way to further address this challenge.

## 7. Conclusions

We propose a local label point correction method for edge detection and image segmentation, which is the first benchmark for label correction based on annotation points. Our LLPC can improve the edge localization accuracy and mitigate labeling error from different annotators in manual annotation. Only three parameters need to be set in our LLPC, but using the label corrected by our LLPC to train multiple networks can yield 30–40% AP improvement. Besides, we construct a largest overlapping cervical cell edge detection dataset based on our LLPC, which will greatly facilitate the development of overlapping cell edge detection. In future work, we plan to develop a label point correction method with local adaptive parameter adjustment.

## Data Availability Statement

The datasets presented in this study can be found in online repositories. The names of the repository/repositories and accession number(s) can be found at: https://github.com/nachifur/LLPC.

## Author Contributions

JL: conceptualization, methodology, software, validation, writing—original draft, and visualization. HF: investigation, resources, writing—review and editing, supervision, project administration, and funding acquisition. QW: writing—review and editing. WL: investigation. YT: writing—review and editing and supervision. DW: investigation, resources, and data curation. MZ and LC: investigation and resources. All authors contributed to the article and approved the submitted version.

## Funding

This work was supported by the National Natural Science Foundation of China (61873259, 62073205, and 61821005), the Key Research and Development Program of Liaoning (2018225037), and the Youth Innovation Promotion Association of Chinese Academy of Sciences (2019203).

## Conflict of Interest

The authors declare that the research was conducted in the absence of any commercial or financial relationships that could be construed as a potential conflict of interest.

## Publisher's Note

All claims expressed in this article are solely those of the authors and do not necessarily represent those of their affiliated organizations, or those of the publisher, the editors and the reviewers. Any product that may be evaluated in this article, or claim that may be made by its manufacturer, is not guaranteed or endorsed by the publisher.

## Footnotes

^1^ https://github.com/Lyken17/pytorch-OpCounter
